# Prevalence and grade of RLS in migraine: A prospective study of 251 migraineurs by synchronous test of c-TTE and c-TCD

**DOI:** 10.1097/MD.0000000000024175

**Published:** 2021-01-29

**Authors:** Qiuxia Zhao, Rong Liu, Jun Zhou, Zhizhi Dong, Yue Chen

**Affiliations:** The First College of Clinical Medical Science, China Three Gorges University; Ultrasound Department of Yichang Central People's Hospital, Yichang, China.

**Keywords:** contrast transcranial doppler ultrasonography, contrast transthoracic echocardiography, migraine, patent foramen ovale, right-to-left shunt

## Abstract

**Background:**

Right-to left shunt (RLS) is regarded as a risk factor resulting in migraine, but the relevance between the RLS and migraine remains controversial. This paper aims at investigating the prevalence and RLS grade of patent foramen ovale (PFO) in cases of migraine (including migraine with and without aura) and evaluate the relationship between PFO and migraine.

**Methods:**

Synchronous test of contrast transthoracic echocardiography and contrast transcranial Doppler ultrasonography was performed in 251 cases of migraine, which contains 62 cases of migraine with aura (MA) and 189 cases without aura (MO) and 275 healthy adults. Among these cases, 25 cases with migraine and 14 healthy adults were evaluated through transesophageal echocardiography.

**Results:**

(1). The prevalence of permanent RLS, total RLS, and large RLS in migraine was 11.16%, 39.04%, and 17.13%, respectively, which was significantly higher than that of the controls (*P* = .042, <.001, and.001, respectively). (2). Permanent RLS was detected as 7.93% of the cases in MO, 20.96% in MA, and 6.18% in controls. Total RLS was detected as 35.98% of the cases in MO, 48.38% in MA, and 23.64% in controls. Large RLS was detected as 13.76% of the cases in MO, 27.41% in MA, and 7.27% in controls. Compared with controls, the positive rate of total RLS and large RLS in MO increased (*P* = .004 and.022, respectively), the that of permanent RLS, total RLS, and large RLS in MA also increased (*P* < .001 for each of the comparisons). The positive rate of permanent RLS and large RLS in MA was remarkably higher than that in MO (*P* = .005 and.013, respectively). (3) The presence of large-size PFO (≥2.0 mm) of migraine showed higher than that of the controls (*P* = .048).

**Conclusions:**

PFO is associated with the migraine (especially with aura), when it is permanent RLS, large RLS, and large-size PFO (≥2.0 mm).

## 1 Introduction

The foramen ovale is an important fetal structure, which closes after birth in most individuals but remains open as a patent foramen ovale (PFO) in approximately 25% of the healthy people.^[1]^ PFO is the most common right-to left shunt (RLS), accounting for about 95% of all RLS.^[2]^ The presence of PFO has been pointed out to be strongly related to various disease processes, which includes cryptogenic stroke, transient ischemic attack, migraine headaches, peripheral arterial embolism, platypnea-orthodeoxia syndrome, and decompression sickness.^[3,4]^ Characterized by moderate or severe headache attacks and reversible neurological and systemic symptoms, migraine is a chronic neurological disorder, which is one of the most prevalent and disabling medical illnesses all over the world.^[5]^ Migraines influence about 13% of the population aged 20 to 64, with one third of migraineurs suffering migraine with aura (MA).^[6,7]^ Furthermore, it appears to be more severe widespread in women.^[8]^ In a meta-analysis, PFO is associated with a 2.5-fold increase in the prevalence of migraine and a 3.4-fold increase in that of MA.^[9]^ Hence, Identifying PFO in migraine cases has great significance. Previous studies have found that contrast transthoracic echocardiography (c-TTE) and contrast transcranial Doppler ultrasonography (c-TCD) are simple, repeatable, and commonly used screening methods, and both positive results can enhance the diagnostic value of PFO.^[10]^ The purpose of this study is to evaluate the prevalence and shunt grade of PFO through synchronous test of c-TTE and c-TCD, and to investigate the correlation between PFO and migraine.

## 2 Methods

### 2.1 Study design

According to the International Classification of Headache Disorders III- beta,^[11]^ 251 cases diagnosed with migraine (aged 14 –74) were recruited from January 2018 to August 2019 at The First College of Clinical Medical Science, China Three Gorges University. Based on a questionnaire, the detailed clinical medical history of these cases was recorded through face-to-face interviews. The first part of the questionnaire, experienced neurologist collected fundamental information such as name, gender, age, height, body weight, and education level, smoking, hypertension, diabetes, dyslipidemia, high D-dimer, silent brain infarcts, and deep white matter lesions. The second part of the questionnaire captured the clinical characteristics of the diagnostic criteria of MA and migraine without aura (MO), and then cases with migraine were classified into 2 subgroups: MA (62 cases) and MO (189 cases). Also, 275 healthy controls (aged 14–81 years) were accrued. However, cases with carotid artery plaque, congenital heart disease, rheumatic heart disease, aortic dissection, malignant arrhythmia, hypercoagulability, and other possible cerebrovascular incidents were excluded. Without being aware of the patient's disease condition, 2 experienced ultrasound technicians implemented the synchronous detection of c-TTE and c-TCD. This study was authorized by the ethics committee (No, HEC-KYJJ2020-002-01). All cases provided informed consent to participate in the study.

### 2.2 Synchronous test of c-TTE and c-TCD

The test was performed with transthoracic echocardiography (TEE, Philips EPIQ7C, China, the probe model X5-1, frequency 1.0∼5.0 MHz) and transcranial Doppler (TCD, Delica 9PB, China, the probe frequency 2 MHz). In this test, participants lay down comfortably in the left lateral position, who were connected to ECG leads. Single channel TCD and double depth monitoring were selected, and the middle cerebral artery was observed through the right temporal bone window. The test was conducted during normal breathing and subsequently with Valsalva maneuver (VM). The contrast agent was produced with 8 mL saline solution, 1 mL air, and 1 ml participant's blood, which was vigorously mixed within the two 10 mL syringes via a 3-way stopcock at least 30 times and then rapidly injected into the right antecubital vein. The first injection was performed during the normal respiration. During VM, the contrast agent was injected 5 seconds before the start of VM. This was produced by the cases blew into a small soft plastic tube connected to the manometer device, and then the cases started the VM at the command of the inspector and held it for 5 seconds. The effective VM was assessed by monitoring the peak flow velocity of the middle cerebral artery. Doppler spectrum was decreased by at least 25%, and the manometer device reached and maintained a pressure of 40 mm Hg.^[12]^ When TTE detected micro-bubbles (MES) in the left atrium within 3 to 5 cardiac cycles, and TCD detected more than 1 MES appeared within 10 seconds after VM was detected, the c-TCD results were regarded as positive. It is assumed that cardiac RLS passes through the PFO when both of c-TTE and c-TCD are positive.

### 2.3 TEE

**Figure 1:**
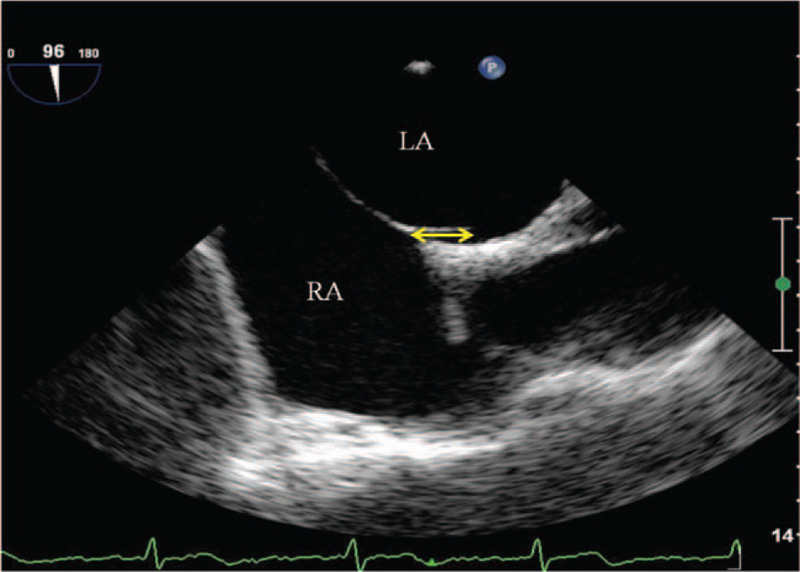
The septum primum against the septum secundum by transesophageal echocardiography. LA = left atrium, RA = right atrium.

Philips iU22 (the probe model S7-2, frequency 3.5∼7.0 MHz) was utilized. The cases were fasting (abstaining from all food and caloric drink) for 4 hours, and oral dyclonine hydrochloride mucilage was taken 10 minutes before the examination. Then the operator was inserted by the operator about 30 to 35 cm away from the incisor. The integrity of the foramen ovale flap was observed at rest, by looking at whether there were fissures between the septum primum against the septum secundum, and whether there were color shunt observed by color Doppler. The height of PFO was measured by the maximum separation between the septum primum and septum secundum at the end of the systole, and a height ≥2 mm was defined as a large-size PFO.^[13]^ The length of PFO tunnel was measured by the maximum overlap between the septum primum and septum secundum and a length ≥10 mm was defined as long-tunnel PFO^[14]^ (Fig. 1).

### 2.4 Image analysis

**Figure 2:**
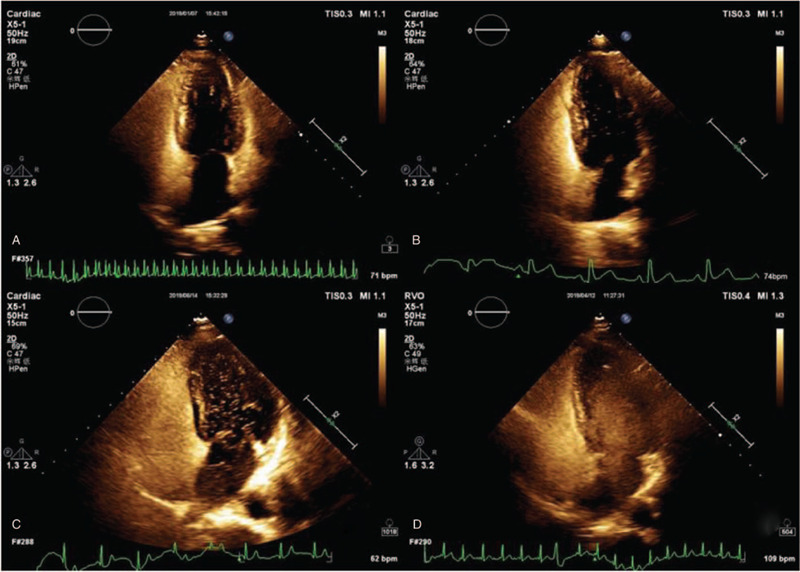
Quantification of right-to left shunt by contrast transthoracic echocardiography. (A): grade 0, no occurrence of micro-bubbles. (B): grade I, 1∼10 micro-bubbles. (C): grade II, 11∼25 micro-bubbles. (D): grade III, over 25 micro-bubbles or left atrium nearly filled with micro-bubbles or left atrial opacity.

**Figure 3:**
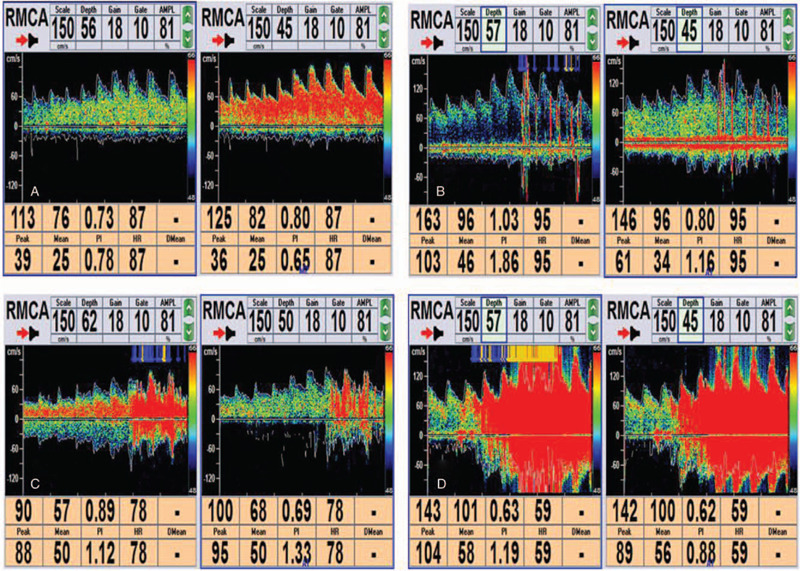
Quantification of right-to left shunt by contrast transcranial Doppler ultrasonography. (A): grade 0, negative. (B): grade I, 1≤ MES ≤10. (C): grade II, MES >10 and no curtain. (D): grade III, curtain. MES = micro-bubbles signal,

According to the grading standards established by the Chinese College of Cardiovascular Physicians,^[15]^ the degree of shunt in c-TTE was quantified based on detected micro-bubbles in the left atrium: grade 0 = no occurrence of micro-bubbles; grade I = 1 to 10 micro-bubbles; grade II = 11 to 25 micro-bubbles; grade III = over 25 micro-bubbles or left atrium nearly filled with micro-bubbles or left atrial opacity (Fig. 2). According to the number of MES, the grade of c-TCD was classified as follows: grade 0 = negative; grade I = 1≤ MES ≤10; grade II = MES >10 and no curtain; grade III = curtain^[16]^ (Fig. 3). Grade I is classified as small shunt, grade II as moderate shunt, and grade III as large shunt. Total RLS includes permanent RLS (RLS also occurred during rest) and provoked RLS (RLS occurred only after the VM).

### 2.5 Statistical analysis

All statistical analyses were performed by using SPSS 19.0. Data was represented by (mean ± SD) for continuous variables and as frequency (n) and percentage (%) for categorical variables. The differences between these 2 groups were analyzed by the *t*-test for continuous variables, and the χ^2^-test for categorical variables. Statistical significance was identified as a value of *P* < .05.

## 3 Results

### 3.1 Characteristics of controls and migraine

A total of 251 cases with migraine and 275 healthy volunteers were recruited. The characteristics of participants were studied in Table 1, such as male/female, age, BMI, smoking, hypertension, diabetes, dyslipidemia, high D-dimer, silent brain infarcts, and deep white matter lesions. No significant differences were shown between these 2 groups

**Table 1:** Characteristics of study participants.

Characteristics	Controls (n = 275)	Migraine (n = 251)	*P* value
Male/female,n	89/186	86/165	.644
Age, yrs	43.2 ± 13.5	43.0 ± 13.7	.861
BMI, Kg/m^2^	22.74 ± 3.38	23.25 ± 3.33	.445
Smoking, n (%)	16 (5.82)	23 (9.16)	.226
Hypertension, n (%)	29 (10.55)	24 (9.56)	.078
Diabetes, n (%)	7 (2.55)	9 (3.58)	.488
Dyslipidemia, n (%)	5 (1.82)	12 (4.78)	.055
High D-dimer, n (%)	1 (0.36)	2 (0.79)	.510
Silent brain infarcts, n ( (%)	19 (6.91)	29 (11.55)	.065
Deep white matter lesions, n (%)	6 (2.18)	2 (0.79)	.195

Date are presented as mean ± SD or n (%) of patents. BMI = body mass index (=calculated as weight in kilograms divided by height in meters squared).

### 3.2 Comparison of shunt type between controls and migraine

The prevalence of permanent RLS, total RLS, and large RLS in migraine was 11.16%, 39.04%, and 17.13%, respectively, which demonstrated obviously higher than that in the control groups (*P* = .042; *P* < .001; *P* = .001, respectively) (Table 2).

**Table 2:** Comparison of shunt type between controls and migraine.

	Controls (n = 275)	Migraine (n = 251)	*P* value
Permanent RLS, n (%)	17 (6.18)	28 (11.16)	.042
Total RLS, n (%)	65 (23.64)	98 (39.04)	<.001
Small RLS, n (%)	31 (11.27)	32 (12.75)	.602
Moderate RLS, n (%)	14 (5.09)	23 ( (9.16)	.068
Large RLS, n (%)	20 (7.27)	43 (17.13)	.001

Data are presented as n (%) of patents. RLS = right-to-left shunt.

### 3.3 Comparison of shunt type between controls, MO and MA

Among the 251 cases of migraine, 189 cases were in the MO and 62 were in the MA. Permanent RLS was detected as 7.93% of the cases in MO, 20.96% in MA, and 6.18% in controls. Total RLS was detected as 35.98% of the cases in MO, 48.38% in MA, and 23.64% in controls. Large RLS was detected as13.76% of the cases in MO, 27.41% in MA, and 7.27% in controls. Compared with the control, the positive rate of total RLS and large RLS in MO increased (respectively *P* = .004; *P* = .022), and that of permanent RLS, total RLS, and large RLS in MA also increased (respectively *P* < .001; *P* < .001; *P* < .001). The positive rate of permanent RLS and large RLS in MA increased indicated significantly higher than that in MO (respectively *P* = .005; *P* = .013) (Table 3).

**Table 3:** Comparison of shunt type between controls, MO and MA.

	Controls (n = 275)	MO (n = 189)	MA (n = 62)	*P* value^∗^	*P* value^†^	*P* value^‡^
Permanent RLS, n (%)	17 (6.18)	15 (7.93)	13 (20.96)	.402	<.001	.005
Total RLS, n (%)	65 (23.64)	68 (35.98)	30 (48. 38)	.004	<.001	.082
Small RLS, n (%)	31 (11.27)	26 (13.76)	6 (9.67)	.423	.717	.403
Moderate RLS, n (%)	14 (5.09)	16 (8.47)	7 (11.29)	.146	.068	.504
Large RLS, n (%)	20 (7.27)	26 (13.76)	17 (27.41)	.022	<.001	.013

Data are presented as n (%) of patents.

∗ *P* value: Control vs MO.

† *P* value: Control vs MA.

‡ *P* value: MO vs MA. MA = migraine with aura, MO = migraine without aura, RLS = right-to-left shunts.

### 3.4 PFO characteristics of TEE

Twenty-five cases with migraine and 14 healthy adults were evaluated through TEE. Compared with the controls (*P* = .048), the presence of large-size PFO (≥2.0 mm) of migraine increased. No differences existed in the length of PFO and long-tunnel PFO (≥10.0 mm) among these groups (*P* = .199; *P* = .095, respectively) (Table 4).

**Table 4:** PFO characteristics of TEE.

characteristics	Controls (n = 14)	Migraine (n = 25)	*P* value
Large-size, ≥2.0 mm	1 (7.14)	9 (36.00)	.048
Length of PFO, mm	7.25 ± 4.09	9.17 ± 4.05	.199
Long-tunnel PFO, ≥10 mm	2 (14.28)	10 (40.00)	.095

Data are presented as mean ± SD or n (%) of patents. PFO = patent foramen ovale, TEE = transesophageal echocardiography.

## 4 Discussion

RLS is an abnormal pathway between the venous and arterial circulations, which includes both intracardiac and extracardiac RLS. Intracardiac RLS are usually connected with PFO, which has been described as a “back door to the brain.”^[17,18]^ The autopsy study of Hagen et al on 965 normal hearts discovered that PFO possessed a prevalence of 27.3% for all ages.^[19]^ Thus, PFO should still be treated as a normal structural variant even without paradoxical embolism or other discomfortable clinical conditions existed.^[20,21]^ The common detection methods of PFO include c-TTE, c-TCD, and Contrast transesophageal echocardiography (c-TEE), both of which has advantages and limitations in diagnosing a patient with stroke, but in principle, they should be equivalent: they all detect RLS through MES, either visually (TTE and TEE) or by Doppler shift (TCD). Consequently, provided that the technique and visualization are adequate, and the physiological mechanism behind the presence of the shunt (at rest or with the VM) is similar, there should be no significant differences in diagnostic accuracy.^[22]^ C-TEE is considered to be the “gold standard” for diagnosing cardiac RLS.^[1]^ However, it is hard to detect small PFO by TEE, there is difficulty in performing the VM during TEE, especially for elderly patients with serious neurological deficits.^[23]^ Besides, other less invasive detection techniques, such as TTE and TCD, have also been improved, which have become the preferred methods for PFO detection.^[12]^ A total of 769 suspected PFO cases were collected in the previous study, and TEE was adopted as the diagnostic standard, and the diagnostic accuracy of synchronous test of c-TTE and c-TCD was 95.2%.^[10]^ Unfortunately, the study subjects involve indecipherable ischemic stroke, transient ischemic attack, migraine, and vertigo. Moreover, independent analyses of migraine patients were not available.

The pathogenesis of migraine is complicated, which is accompanied by various risk factors. Studies have revealed that age, smoking, hypertension, hyperlipidemia, and diabetes could increase the risk of migraine, possibly because of increased risk factors of blood vessels, blood hypercoagulability, and vascular dysfunction.^[8,24]^ In addition, current evidence demonstrated that obesity could increase the risk and severity of migraine, especially for women in childbearing age. This may be associated with several hormones such as leptin and adiponectin, and some migraine patients may be able to relieve migraine symptoms through weight loss interventions.^[8]^ The relationship between migraine and subclinical brain ischemic lesions including silent brain infarctions and white matter hyperintensities, is complicated and disputed. Sas et al^[25]^ proposed that migraine and stroke may have a common pathogenesis, both of which may be accompanied by neuronal dysfunction and neuronal vulnerability, thus resulting in neurodegeneration and apoptosis. In conclusion, comparable characteristics of the studied participants play an important role in this study.

At present, the relationship between RLS and migraine is still being debated, the prevalence of RLS between both migraineurs and healthy individuals varies. In 1998, Del Sette et al^[26]^ pointed out that 18 (41%) of these 44 cases with migraine presented RLS compared with 8 (16%) of 50 controls (*P* < .005), however, the limitation of this study was that the volume of the sample is small. In the study of Yang,^[27]^ 217 consecutive migraine in total were contained, which found that the prevalence of RLS showed significantly higher in Chinese migraineurs than that in healthy controls. Meanwhile, it revealed that the detection rates of migraine and control group were 28.0% and 44.2%, respectively, and the prevalence of RLS was 66.1% and 36.1% in the MA and MO, respectively, which indicated significantly higher than that of the healthy group and higher than the detection rate in this study. In this study, the prevalence of permanent and total RLS in migraine showed higher than that of controls. Additionally, compared with controls, the positive rate of RLS in both MA and MO increased (*P* < .05). This coincides with the study of Yang et al,^[28]^ which proposed that PFO-related may be associated with migraine (MA and MO). However, Yang only channeled c-TCD into evaluating the positive rate of RLS, which was difficult to determine the anatomic origin of intracranial MES, namely, it can not exclude extracardiac RLS, and to some extent it affects the accuracy of the results.^[12]^ However, c-TTE can offset the shortcomings of c-TCD, which has advantages in judging intracardiac RLS, so the results are expected to be more accurate.

The mechanism of PFO that causes migraine is unknown. The possible causes are coughing, straining to defecate, the VM, and lifting heavy objects which can lead to right atrial pressure raised exceeding the left atrial pressure, thus making it easier for RLS to pass through a PFO. MES and metabolite products from venous circulation enter into the intracranial artery, generating brain stimulation.^[12]^ Nozari et al^[29]^ observed in mice experiments that small particulate or air emboli injected into the carotid artery could induce a cortical spreading depression without causing ischemia. This study revealed that abnormal microembolism and ischemia may be the trigger factor for migraine.^[29–31]^ Furthermore, the correlation between PFO and migraine may be related to genetics. A recent report pointed out that the occurrence of atrial shunt was in line with autosomal dominant inheritance to some families with aura migraine.^[32]^

In this study, compared with controls, the proportion of large shunt in the MA group and the MO group rise (especially in MA), which indicated that the correlation between PFO and migraine may be related to large shunt. Larger RLS may increase the possibility of migraine, which suggests that a “neuronal threshold” exceeding this threshold will trigger migraine. Jesurum et al^[33]^ reported a follow-up study of 67 migraineurs who had migraine symptoms after transcatheter PFO closure, which adopted migraine relief (>50% reduction in frequency) as the endpoint. It turned out that migraineurs with aura were 4.5 times more likely to relieve migraine than those without aura. Even though some patients have RLS shunt, no statistically significant difference was represented in migraine symptom relief between the complete and the incomplete group (77% vs 83%, *P* = .76). In a world, migraine may be relieved despite of residual RLS after transcatheter PFO closure, which may indicate that the RLS burden is reduced below a neuronal threshold that triggers migraine. Some scholars have stated that migraineurs with RLS were connected with impairment of dynamic cerebral autoregulation. Guo et al^[34]^ separated 66 migraine cases into the RLS group (n = 30) and the non-RLS group (n = 36). It could be observed that phase difference of patients in the RLS group showed significantly lower than those in the non-RLS group (*P* < .001), Besides, the PD in the large RLS group was significantly lower than that of the small RLS group (*P* < .01) and non-RLS group (*P* < .001). Dynamic cerebral autoregulation was impaired in migraineurs with large RLS, which may represent a potential mechanism linked to RLS and migraine. Transcatheter PFO closure has recently become an effective therapy to improve migraine symptoms and reduce ischemic events. It is essential to analyze PFO characteristics and identify high-risk PFO. In contrast to controls, the prevalence of large-size PFO (≥2.0 mm) increased (*P* = .048), but the length of PFO and long-tunnel PFO (≥10.0 mm) showed no difference among these groups (respectively, *P* = .199, *P* = .095). It was proved that PFO may cause migraine related to the large-size PFO, which may be because of the increasing increased risk of paradoxical embolism in large PFO.^[35]^

These discoveries of a possible connection between migraine and PFO is still controversial. Generally, embolic events showed an unpredictable hemispheric distribution, while migraine pain is typically lateralized, often periodic and predictable like menstrual migraine. Furthermore, the headache symptoms of patients with PFO combined with migraine can be eased with age.^[32]^ Whether the relationship between PFO and migraine is causal or symbiotic remains to be researched.

Our study may have some limitations: first of all, some of the researched subjects were outpatients, which may have bias in the selection. Secondly, in this research, the healthy adults tried to be chosen to match the migraine group with respect to characteristics and no significant differences in characteristics between the 2 groups, but other indicators like blood biochemistry, drug use, and treatment status are not available to be obtained. Lastly, the diagnostic method of PFO is TEE. Due to the invasive examination and difficulty with VM, only some patients have finished TEE.

## 5 Conclusion

PFO is associated with migraine (especially with aura), when the PFO is permanent RLS, large RLS, and large-size PFO (≥2.0 mm).

## Author contributions

**Data curation:** Qiuxia Zhao, Zhizhi Dong.

**Formal analysis:** Qiuxia Zhao, Rong Liu.

**Funding acquisition:** Qiuxia Zhao.

**Investigation:** Qiuxia Zhao.

**Methodology:** Yue Chen, Qiuxia Zhao, Jun Zhou, Zhizhi Dong.

**Project administration:** Rong Liu, Jun Zhou.

**Resources:** Rong Liu.

**Writing – original draft:** Qiuxia Zhao, Yue Chen.

**Writing – review & editing:** Qiuxia Zhao, Jun Zhou, Rong Liu.

## Abbreviations

Abbreviations: c-TCD = contrast transcranial Doppler ultrasonography, c-TTE = Contrast transthoracic echocardiography, MA = migraine with aura, MCA = middle cerebral artery, MES = micro-bubbles signal, MO = Migraine without aura, PFO = Patent foramen ovale, RLS = right-to left shunt, TEE = transesophageal echocardiography, VM = Valsalva maneuver.
